# Standardized *in vitro* bleeding tests in a non-coated novel hybrid prosthesis for frozen elephant trunk demonstrates minimal oozing during full heparinization, supported by clinical data

**DOI:** 10.3389/fcvm.2023.1303816

**Published:** 2023-12-14

**Authors:** Heinz Jakob, Timo Leins, Meltem Avci-Adali, Christian Schlensak, Daniel Wendt, Yatin Mehta, Matthias Thielmann, Klaus Görlinger, Suk-Won Song, Konstantinos Tsagakis

**Affiliations:** ^1^ Diagnosticum Mülheim, Mülheim, Germany; ^2^ Department of Thoracic- and Cardiovascular Surgery, West German Heart and Vascular Center, Essen, Germany; ^3^ Medira GmbH, Balingen, Germany; ^4^ JOTEC-Artivion, Hechingen, Germany; ^5^ Department of Thoracic and Cardiovascular Surgery, University Hospital Tuebingen, Tuebingen, Germany; ^6^ CytoSorbents Europe, Berlin, Germany; ^7^ Medanta Institute of Critical Care and Anesthesiology, Medanta - The Medicity, Gurgaon, India; ^8^ Tem Innovations GmbH, Munich, Germany; ^9^ Department of Thoracic and Cardiovascular Surgery, College of Medicine, Ewha Womans University, Seoul, Republic of Korea

**Keywords:** dissection, frozen elephant trunk (FET), *in vitro*, bleeding, aortic surgery, hemoadsorption

## Abstract

**Introduction:**

Recent reports have questioned the blood impermeability of the novel frozen elephant trunk (FET) device E-vita Open NEO^©^ (EO-NEO). Therefore, standardized *in vitro* bleeding tests using porcine heparinized blood were performed, as well as stress testing on the blood tightness of the collar suture line, to investigate this observation.

**Material and methods:**

EO-NEO prostheses were examined *in vitro* for blood permeability in three test series. Initially, antegrade perfusion with heparinized porcine blood [activated clotting time (ACT) of 500 s, with a 60 min duration] was performed, followed by ante/retrograde testing via the EO-NEO side port. Testing of the collar suture line under a tension of 10 Newton (N) within a suspension device (blood pressure 120 mmHg, ACT of 560 s, 1 min duration) was carried out with the suture material force fiber white (FFWs) yarn, using standard fixation (5 stitches/cm), FFWh yarn in hemostatic fixation (15 stitches/cm), and flow weave yarn (FWYh).

**Results:**

Blood permeability testing of EO-NEO through the prosthetic lumen or via the side port demonstrated minor leakage without statistical difference between the standard and hemostatic suture lines or suture materials used, or positioning on the crimped or tapered portion (*p* > 0.05). The specific collar anastomosis testing demonstrated leakage volumes of 140 ml/min for FFWs vs. 16 ml/min for FFWh (*p* = 0.02), vs. 9 ml/min with the FWYh (*p* = 0.01).

**Conclusion:**

Different blood leakage tests showed minimal oozing and no difference in blood loss through the fabric and different collar suture lines, but unphysiological pressurized retrograde perfusion of the collar region showed significantly less leakage using FWYh and FFWh, prompting production modification of EO-NEO. Clinical results confirmed low blood loss using this novel FET device.

## Introduction

In 2005, the first commercially available hybrid aortic arch, the descending aorta stent graft, was introduced by us, the authors, in collaboration with JOTEC GmbH in Hechingen, Germany (1). The woven prosthesis was non-coated to be intussusceptive into the stented portion, which itself was compressed within a polyester sleeve and mounted on the application system. This offered the possibility of introducing the hybrid prosthesis into the descending aorta through the opened arch and releasing the stented portion. Thereafter, the anastomosis with the distal arch in zone 3 prior to unfolding the intussuscepted arch portion, by retracting it into the arch position for anastomosing with the head vessels and the ascending aortic graft, was completed. Another important aspect of this new concept was the avoidance of bovine-origin collagen coating. It is known that bovine coating might theoretically be associated with the potential transfer of bovine spongiform encephalopathy (BSE, mad cow disease). Therefore, the graft fabric required meticulous pre-sealing with human-derived fibrin glue (Tisseel^©^, Baxter, Germany) for blood impermeability during full heparinization (2). To simplify its application, the later named E-vita Open Plus^©^ (EO PLUS) graft was manufactured by a tighter weaving process and introduced in 2008 with a significant reduction in oozing through the graft’s pores during full heparinization, which could be proven in experimental and clinical studies (3). With the introduction of the CE-marked EO-NEO in 2020, the tightly woven material was maintained with the principle of non-precoating (4). This applies to all three new variations of frozen elephant trunk (FET) prostheses—straight, branched, and trifurcated versions—to offer easier surgical adaptation to any kind of pathoanatomic situation.

A casuistic report by Czerny et al. on three massive oozing incidents, and a consecutive *in vitro* study by Tan and Bashir, as well as a systematic coagulopathy review by Bashir and colleagues, raised concerns (5–7). Those reports, as well as individual case reports, prompted us, in collaboration with the JOTEC CryoLife/ARTIVION company and the Clinical Research Laboratory of Department of Cardiac, Thoracic and Vascular Surgery, University Hospital Tübingen, to scrutinize those observations by performing *in vitro* experiments with the novel hybrid prosthesis. The following questions had to be addressed: Where does the reported casuistic bleeding come from? Is there a mechanical defect? Finally, the results were related to the clinical experiences reported thus far.

## Material and methods

**Figure 1:**
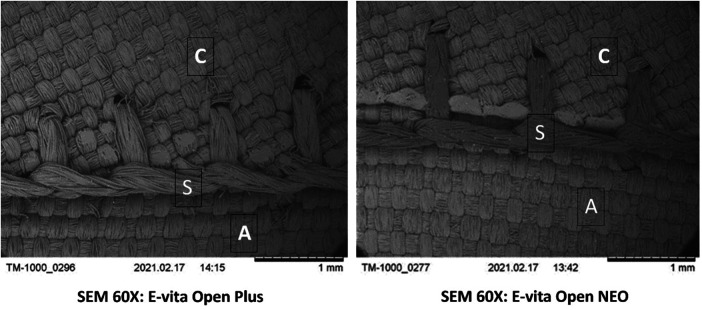
SEM demonstration of suture hole stress testing (10 N) on a Hitachi TM-1000 scanning electron microscope. C, collar; S, standard suture line for collar fixation; A, arch portion fabric.

**Figure 2:**
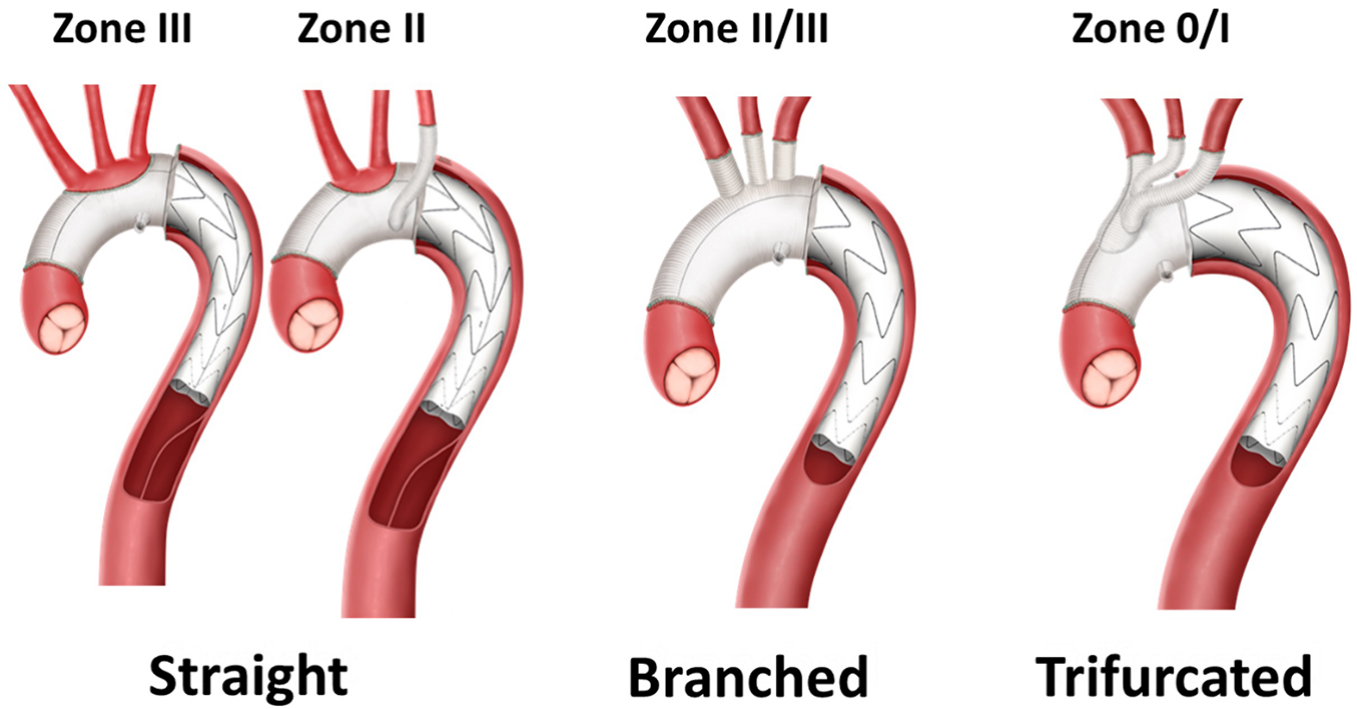
E-vita open - NEO© (CE-mark 3/2020) in its three variations. Straight, branched, and trifurcated including a perfusion port to accomodate implantation at all pathoanatomic situations. Anastomotic options according to Ishimaru zones 0–III.

The prerequisite basic facts are: there is no difference in the fabric between EO PLUS and EO-NEO. The fabric tubes for EO Plus and EO-NEO are produced on the same loom, the collar material is identical (laser cut out of the same fabric tube), and the suture material has the same specification (5.0 braided surgical suture) in both. For basic orientation, a TM-1000 (Hitachi, Tokyo, Japan) scanning electron microscope (SEM) was used to study suture hole width resulting from the manufacturing after a tension of 10 N was placed on the collar anastomosis of the native EO-NEO and was compared with EO PLUS at a 60× magnification and preceded blood stress testing (Figure 1). The test circuit consisted of a roller pump connected to silicone tubing, porcine blood bag, manometer, and flow probe. The silicone tubing was partitioned into four branches, all connected to four containment chambers, in which the prostheses were mounted to sample blood loss over defined time slots. The EO-NEO devices tested are depicted in Figure 2. Owing to the COVID-19 pandemic lockdown in 2021/2022, the originally planned use of human blood had to be abandoned. Thus, easily available fresh heparinized porcine blood had to be used.

### First test set up: whole prosthesis blood permeability testing by antegrade perfusion

**Figure 3:**
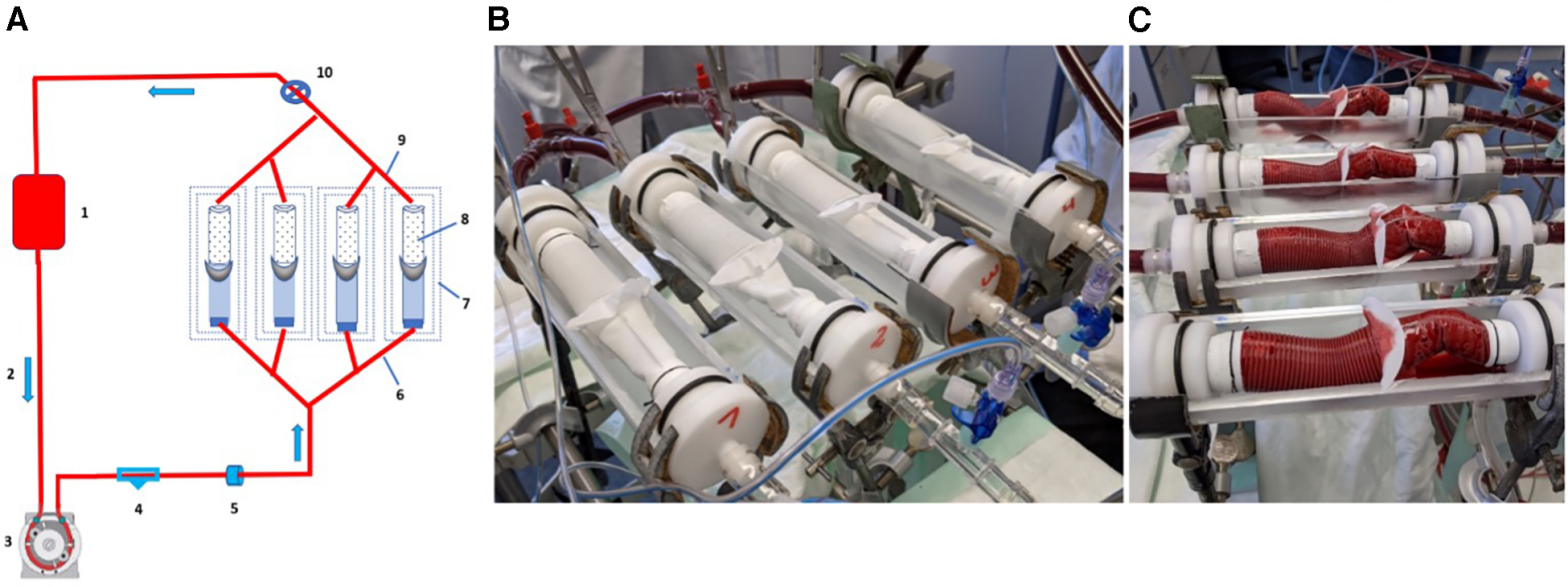
(**A**) Schematic drawing of antegrade perfusion with fresh heparinized porcine blood through EO-NEO. 1. Porcine blood bag; 2. 3/8" Slicone tubing – flow direction; 3. Roller pump; 4. Manometer; 5. Flow probe; 6. Branched inflow into EO NEO straight, no side arm; 7. Containment chamber; 8. EO NEO Stented portion; 9. Prosthetic outflow; 10. Blood pressure regulation with clamp. (**B**) Experimental set up. (**C**) Perfusion with heparinized porcine blood. Sample 1: FFWh, hemostatic fixation; Sample 2: FWYh, hemostatic fixation; Sample 3: EEs, standard fixation; Sample 4: FFWBs, standard fixation.

The test set up consisted of four in-series placed containment chambers in which four straight full-length EO-NEO prostheses without a perfusion port were clamped in using PTFE bands and silk ties for fixation into inflow and outflow hollow silicone plugs with integrated 3/8' tubing. These were connected with adapters to the tubing circuit with integrated flow probes and manometers (Figures 3A,B).

The collar was expanded with two stay sutures horizontally fixed at the chamber’s upper edges under mild tension. Blood loss was documented in separate measuring cylinders. Two bags of heparinized porcine blood (ACT of 500 s) were used to fill the test circuit and pump flow was started with a conventional roller pump after deairing at a full flow of 2 L/min at 40 mmHg for equilibration within 5 min, followed by the augmentation of blood pressure to 120 mmHg by partial clamping of the tubing for the ensuing 55 min. Oozing through the prostheses’ pores was collected and measured at 5-min intervals for 60 min (Figure 3C).

The prosthetic material consisted of tapered EO-NEO full-length prostheses, and different 5–0 sewing material, with the collar anastomosed either in standard fashion (5 stitches per cm) or hemostatic (15 stitches per cm):

Sample 1: Suture material A (force fiber white, FFWh), hemostatic fixation
Sample 2: Suture material B (flow weave yarn, FWYh) hemostatic fixation
Sample 3: Suture material C (Ethibond Excel, EEs), standard green filament from EO PLUS, standard fixation
Sample 4: Suture material D (force fiber white-blue, FFWBs), standard fixation

### Second test set up: whole prosthesis blood permeability perfused ante-retrogradely via the side port

**Figure 4:**
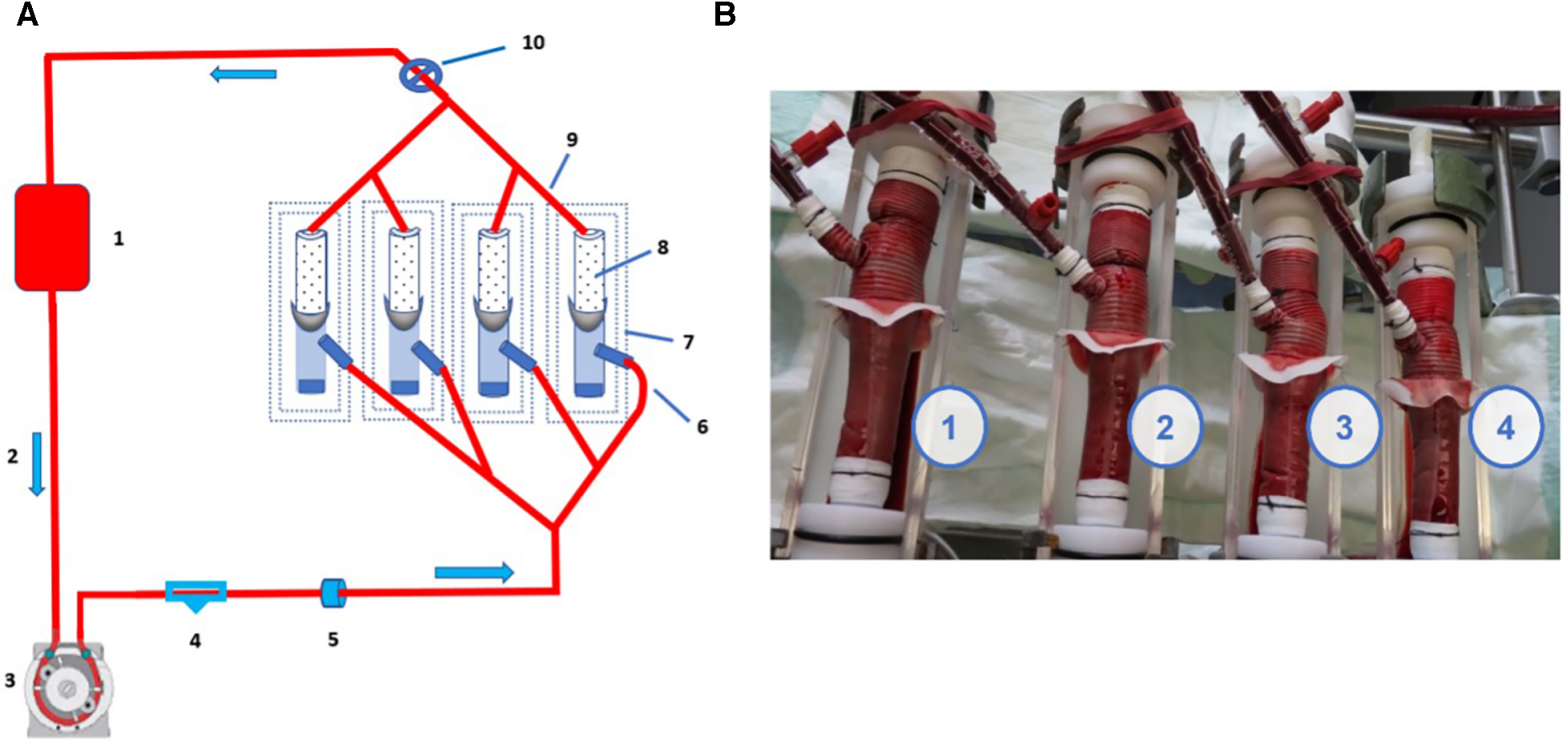
(**A**) Schematic drawing of ante-retrograde perfusion through the side port at the prosthesis’ arch portion. 1. Porcine blood bag; 2. 3/8" Slicone tubing – flow direction; 3. Roller pump; 4. Manometer; 5. Flow probe; 6. Inflow into EO NEO via side arm; 7. Containment chamber; 8. EO NEO Stented portion; 9. Prosthetic outflow; 10. Blood pressure regulation with clamp. (**B**) Perfusion with heparinized porcine blood (ACT of 500). 1. FFWs – Collar at crimp, standard fixation; 2. FWYh – Collar at tapering, hemostatic fixation; 3. FFWs – Collar at tapering, standard fixation; 4. FWYh – Colar at crimp, hemostatic fixation.

To control the suture integrity of the fabric and branches, including the collar and perfusion port, dynamic blood permeability testing was performed as in series 1. The test set up consisted of four in-series placed containment chambers with four EO-NEO prostheses clamped in. The flow rate was set at 2 L/min at a pressure primarily at 40 mmHg for 5 min for equilibration, and thereafter at 120 mmHg. ACT was equilibrated at 500 s. Inflow was directed into the straight EO-NEO prostheses via the side arm perfusion port with the proximal prosthetic end blocked (Figure 4A). Oozing through the prostheses’ pores was collected and measured at 5-min intervals for 60 min after the primary 5-min equilibration phase. To scrutinize whether the position of the collar suture line was more effective either at the crimped arch portion of the EO-NEO or on the tapered portion of the stented part of the prosthesis, the test set up was constructed as follows (Figure 4B):

Sample 1: Collar position moved to the first crimp, suture material A1 (FFWs), standard fixation suture technique
Sample 2: Standard collar positioning at the tapered portion, suture material B1 (FWYh), hemostatic suture technique
Sample 3: Standard collar position at the tapered portion, suture material A2 (FFWs), standard fixation suture technique
Sample 4: Collar position moved to the first crimp, suture material B2 (FWYh), hemostatic suture technique

### Third test set up: simulation of retrograde flow onto the collar only

**Figure 5:**
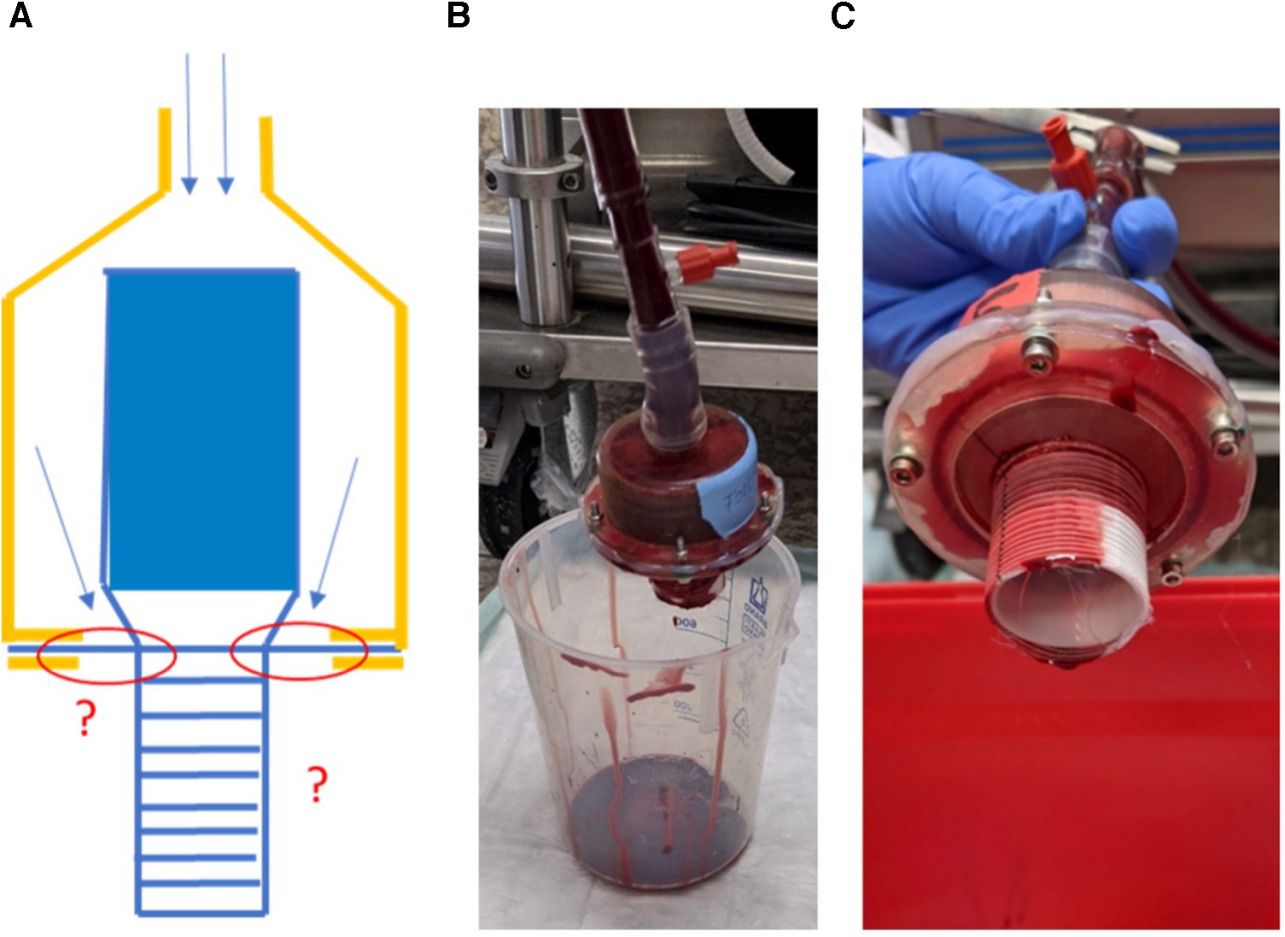
Sole collar blood permeability testing under tension (10 N) with heparinized porcine blood at 120 mmHg for 1 min (ACT of 559 s). (**A**) Schematic drawing of collection vessel with plugged prosthetic lumen (**B**) Leakage measuring unit for collar anastomosis. (**C**) Demonstration of sole collar leaking into collection vessel.

Five hundred milliliters of heparinized (ACT of 560 s) porcine blood was brought through 3/8' silicone tubing vertically mounted and manometrically controlled at a pressure of 120 mmHg for 1 min onto the collar region of three test sets with five samples each of EO-NEO prostheses reduced to a length of 10 cm (crimped status), with the prosthetic lumen obliterated. The collar was put under a tension of 10 N to overemphasize a clinical scenario (Figure 5). The sewing material and sewing technique tested was:

Sample 1: Suture material A (FFWs), standard suture technique
Sample 2: Suture material A (FFWh), hemostatic suture technique
Sample 3: Suture material B FWYh), hemostatic suture technique

The leakage was measured within a collection vessel in milliliters per 1 min.

## Statistical analysis

Data were analyzed using SPSS version 27 software (IBM, Armonk, NY, USA). Leakage volumes (continuous variables) were expressed as mean ± standard deviation (SD) and compared using Student's *t*-test. Clinical data (categorical data) were expressed as the number of patients and frequencies or mean ± standard deviation (SD). *P*-values <0.05 were considered significant.

## Results

**Figure 6:**
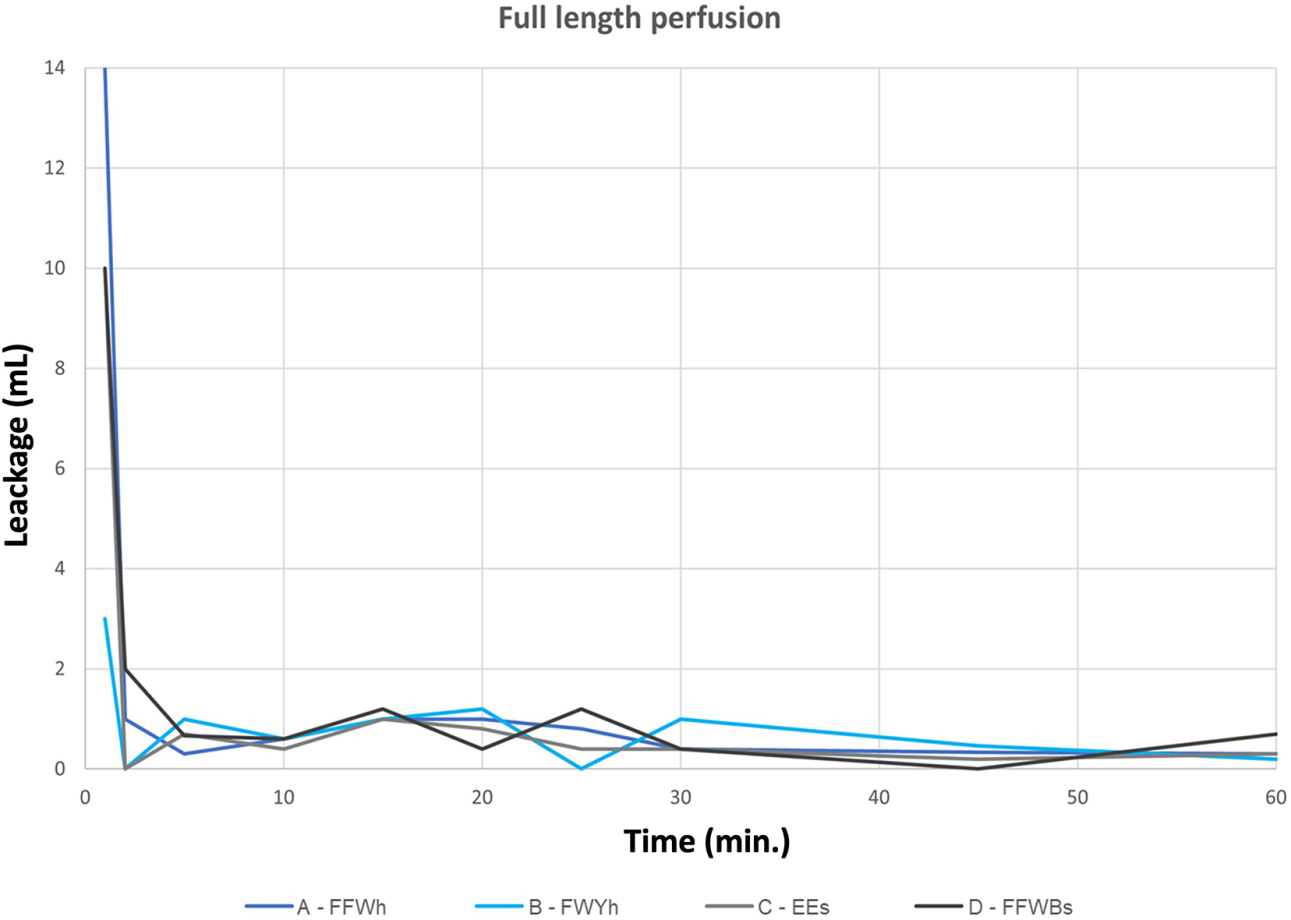
Full-length EO NEO blood permeability testing at 120 mmHg H_2_O over 60 min (ml/min) with different collar suture variants.

SEM analyses and the comparison of suture hole width under a stress tension of 10 N stress did not show any microscopic difference between the classic EO PLUS and EO-NEO fabrics (Figure 1). In the first test series with antegrade perfusion of full-length EO-NEO prostheses without perfusion ports or branches, there was no significant difference (*p* > 0.05) in blood loss by oozing through the prostheses’ pores between the four different suture techniques of collar anastomoses, as demonstrated in Table 1 and Figure 6. Within a few minutes, primary oozing ceased through the prosthetic pores and no difference in collar suture bleeding was observed. After the observation time span, longitudinal transection of the prostheses allowed measurement of the total prosthetic area. Blood loss was marginal (between 0.0021 and 0.0037 ml/cm^2^ per minute) with the four different suture materials regardless of technique (standard or hemostatic) (*p* > 0.05) (Table 1A).

**Table 1:** Blood loss measurements (ml) through full-length EO NEO at 120 mmHg over 60 min after 5 min of equilibration at a pressure of 40 mmHg (ACT of 500 s).

	Leakage [ml]			
Time, [min]	A	B	C	D
5	15.3	4.0	10.7	12.7
10	0.6	0.6	0.4	0.6
15	1.0	1.0	1.0	1.2
20	1.0	1.2	0.8	0.4
25	0.8	0.0	0.4	1.2
30	0.4	1.0	0.4	0.4
35	0.1	0.2	0.1	0.0
40	0.1	0.2	0.1	0.0
45	0.1	0.2	0.1	0.0
50	0.1	0.1	0.1	0.2
55	0.1	0.1	0.1	0.2
60	0.1	0.1	0.1	0.2
Total	19.7	8.7	14.3	16.9

**Table 1A:** Average leakage/oozing results (ml per cm^2^ per minute; ACT of 500 s, full length prostheses).

A	B	C	D
FFWh	FWYh	EEs	FFWBs
0.0033 ml/cm^2^/min	0.0021 ml/cm^2^/min	0.0026 ml/cm^2^/min	0.0031 ml/cm^2^/min

**Figure 7:**
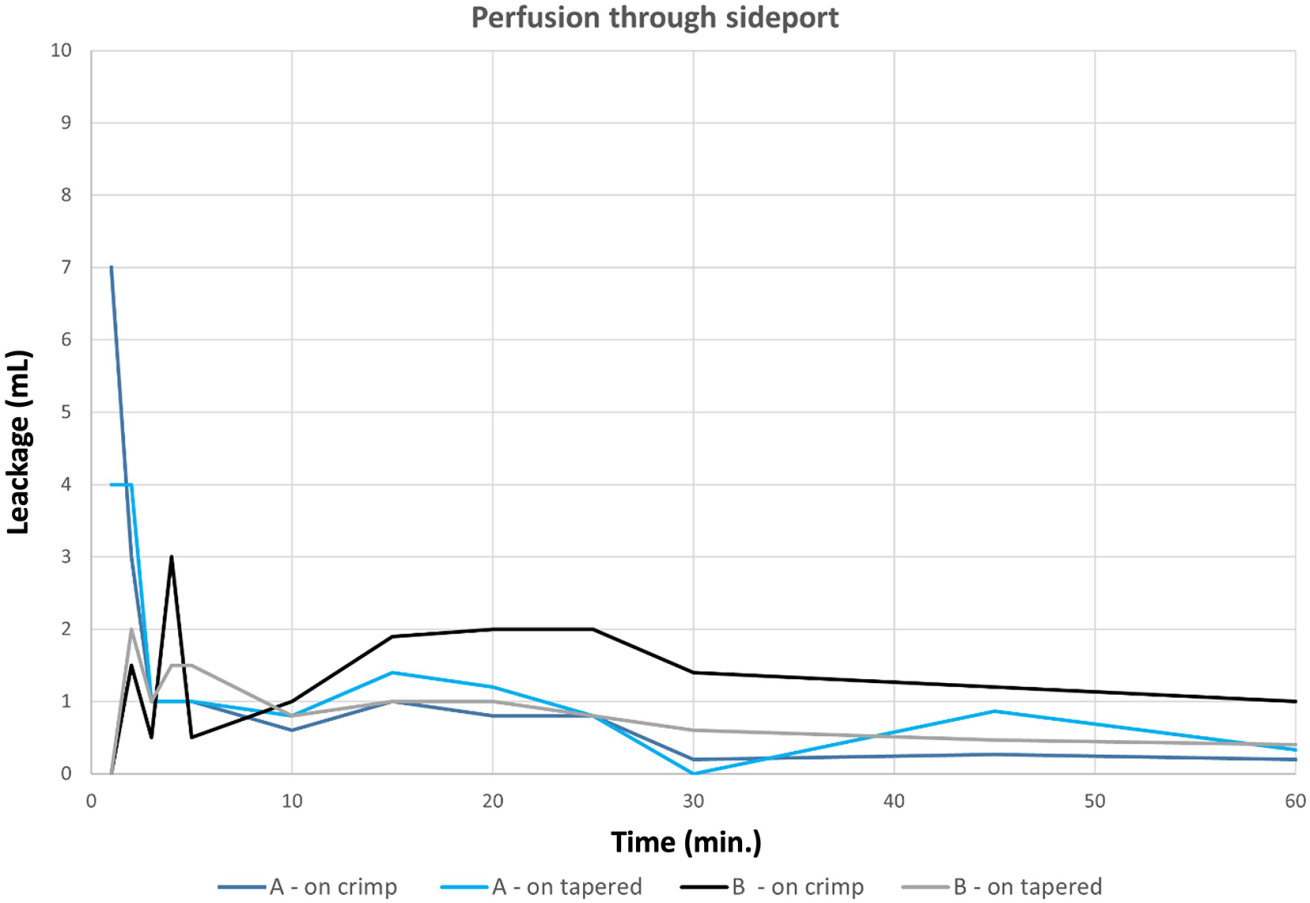
Antegrade perfusion through the side port for 60 min (first 5 min at 40 mmHG, thereafter at 120 mmHg; ACT of 614 s).

In test series 2, ante-retrogradely perfusion via the perfusion port again did not show any significant difference between the location on either the crimped or tapered portion of the EO-NEO, no matter whether the suturing technique was standard or hemostatic, as demonstrated in Table 2 and in the graphic representation in Figure 7 (*p* > 0.05).

**Table 2:** Whole prosthesis blood permeability perfused ante-retrogradely via the side port.

	Leakage [ml]			
Time (min)	A on crimp	A on tapered	B on crimp	B on tapered
5	13	11	5,5	6
10	3	4	5	4
15	5	7	9.5	5
20	4	6	10	5
25	4	4	10	4
30	1	0	7	3
35	2	4.3	6	2.3
40	1	4.3	6	2.3
45	1	4.3	6	2.3
50	1	1.6	5	2
55	1	1.6	5	2
60	1	1.6	5	2
Total	37	50	80	40

Collar anastomosis on the crimped or tapered sections.

**Figure 8:**
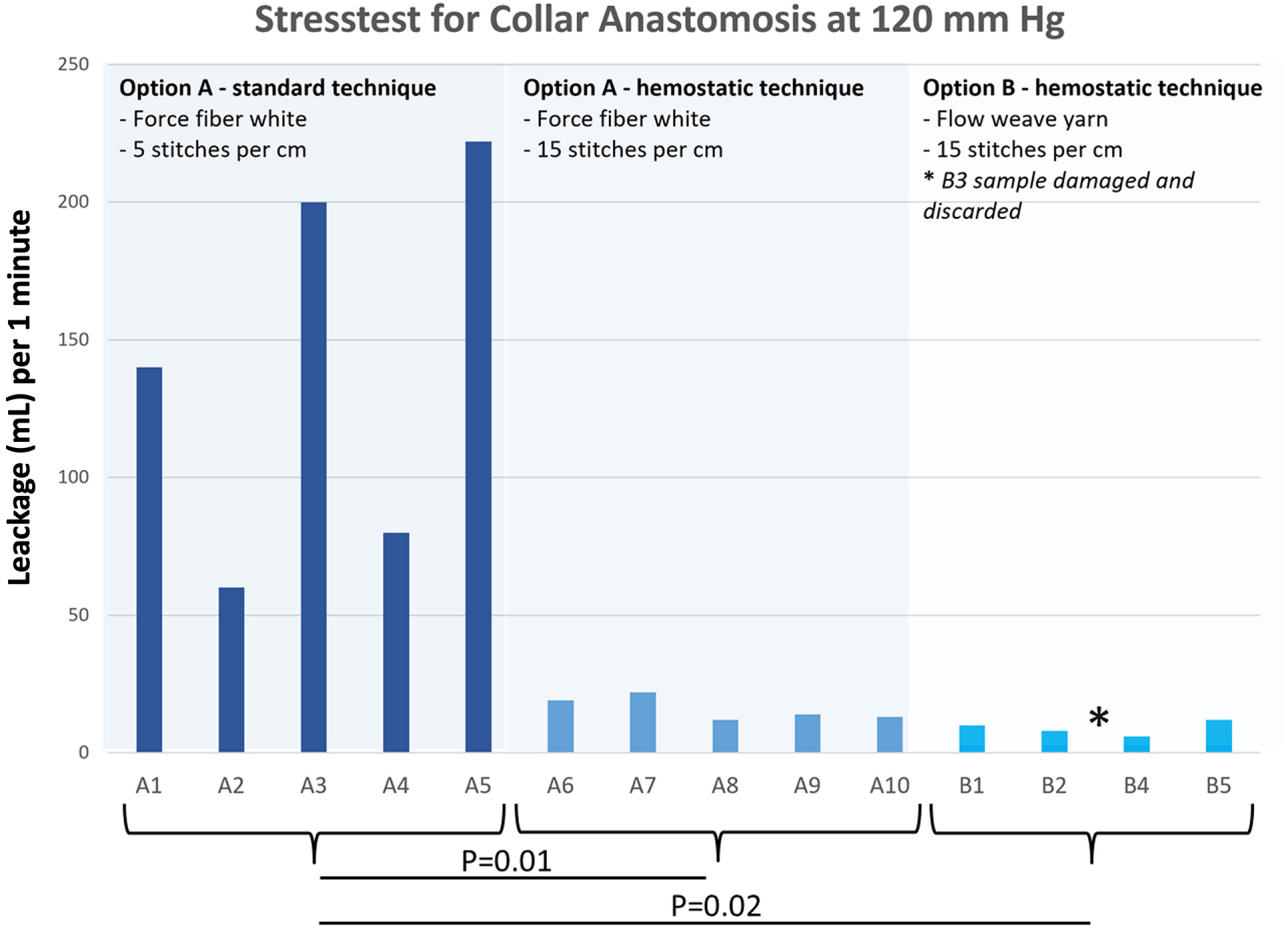
Stress test for collar anastomosis by retrograde pressurized exposure to 120 mmHg for 1 min with heparinized porcine blood (ACT of 559 s).

In test series 3, the unphysiological stress test directing pressurized heparinized porcine blood at 120 mmHg precisely on the expanded collar under a tension of 10 N with five samples each demonstrated significantly less permeability with the hemostatic suture lines for collar fixation than with the standard suture fixation FFWs (mean 140 ml/min compared with 16 ml/min for FFWh, *p *=* *0.02) or with FWYh (mean blood loss 9 ml/min, *p* = 0.01, Figure 8 and Table 3)

**Table 3:** Stress test of collar anastomosis by retrograde pressurized exposure to 120 mmHg for 1 min with heparinized porcine blood (ACT of 559 s).

	Retrograde perfusion of the collar area (1 min, 120 mmHg)	
Sample No.	Suture material fixation standard (5) versus hemostatic (15) stitches/cm	Leakage [ml]
A 1	Force fiber white - standard	140
A 2	Force fiber white - standard	60
A 3	Force fiber white - standard	200
A 4	Force fiber white - standard	80
A 5	Force fiber white - standard	222
A 6	Force fiber white - hemostatic	19
A 7	Force fiber white - hemostatic	22
A 8	Force fiber white - hemostatic	12
A 9	Force fiber white - hemostatic	14
A 10	Force fiber white - hemostatic	13
B 1	Flow weave yarn - hemostatic	10
B 2	Flow weave yarn - hemostatic	8
B 4	Flow weave yarn - hemostatic	6
B 5	Flow weave yarn - hemostatic	12
B 3	discarded	damaged

Clinical data from Essen University Hospital (Table 4) and Ewha Womans University Aorta and Vascular Hospital, Seoul, South Korea (Table 5) confirm low perioperative blood product use due to minor blood loss within 24 hours. The low postoperative re-operation rate for bleeding was based on collar surrounding big bites over a teflon felt supporting close attachment of the aortic wall to the collar site of the EO NEO (Essen), or a double row suture technique (Seoul). All prostheses used had standard collar anastomosis fabrication.

**Table 4:** Clinical results of the university of Essen.

Demographics (May 2021 – May 2022)	Patients (*n* = 56)
Age, years	60.1 ± 11.1
Male	36 (64.3)
Aortic disease	
Acute type A aortic dissection	23 (41.1)
Acute type B aortic dissection	7 (12.5)
Chronic type A aortic dissection	7 (12.5)
Chronic type B aortic dissection	5 (8.9)
Thoracic/-abdominal aortic aneurysm	14 (25)
Procedural details	
Aortic cross-clamp time, [min]	129.3 ± 34.1
Cardiopulmonary bypass time, [min]	232.2 ± 54.7
Hypothermic circulatory arrest time, [min]	46.6 ± 14.0
Antegrade cerebral perfusion time, [min]	68.2 ± 23.5
Operative outcomes	
Transfusions, units	
Packed red blood cells	7.0 ± 4.9
Fresh frozen plasma	–
Platelets	3.0 ± 1.2
Cryoprecipitate	–
Fibrinogen, *g*	8.2 ± 4.6
Prothrombin complex concentrate (PCC)	3,803.5 ± 2,605.3
Perioperative outcomes	
Re-operation due to bleeding	3 (5.4)
24 h drainage volume, ml	1,022.1 ± 461.9
Hospital stay, days	17.3 ± 10.7
In-hospital mortality	6 (10.7)
30-day mortality	5 (8.9)

Data presented as mean ± SD or a number (%).

**Table 5:** Clinical results university of Ewha womans university.

Demographics (April 2021 – December 2022)	Patients (*n* = 139)
Age, years	65.3 ± 14.2
Male	105 (75.5)
Aortic Disease	
Acute type A aortic dissection/IMH	60 (43.2)
Acute type B aortic dissection/IMH	14 (10.1)
Chronic type A aortic dissection	4 (2.9)
Chronic type B aortic dissection	11 (7.9)
Complex aortic aneurysm	50 (36.0)
Procedural details	
Aortic cross-clamp time, [min]	123.8 ± 30.6
Cardiopulmonary bypass time, [min]	153.8 ± 36.0
Hypothermic circulatory arrest time, [min]	54.3 ± 10.4
Antegrade cerebral perfusion time, [min]	124.4 ± 30.0
Operative outcomes	
Transfusions, units	
Packed red blood cells	1.2 ± 1.7
Fresh frozen plasma	5.5 ± 1.6
Platelets	11.7 ± 4.0
Cryoprecipitate	0.8 ± 2.9
Fibrinogen, *g*	–
Perioperative outcomes	
Re-operation due to bleeding, *n* (%)	5 (3.6)
24 h drainage volume, ml	819.7 ± 521.4
Hospital stay, days	17.1 ± 15.9
In-hospital mortality	3 (2.2)
30-day mortality	1 (0.7)

Data presented as mean ± SD or number (%).

## Discussion

The FET concept certainly represents an important step forward, aimed at reducing the previous two or even three step approaches needed to cope with multilevel thoracic aortic disease, but it does require advanced surgical skills, especially when all-comer pathologies, including emergency situations, are to be handled (8). Currently, four devices are available globally: the Cronus open (MicroPort Medical, Shanghai, China) from 2004 used mainly in China (9); the Frozenix-J graft (LifeLine, Tokyo, Japan) used mainly in Japan from 2014 (10); and the E-vita open (plus) graft from 2005 (1) and the Thoraflex hybrid graft (Vascutek, Inchinnan, United Kingdom) from 2010 (11), both used mainly in the western world. A first report of a significant improvement regarding long term freedom from reoperation after acute type A aortic dissection surgery, in FET vs. proximal repair, was reported by us in 2020 (12). Based on the same manufacturing process as for the EO PLUS, the device becomes blood impervious after a brief period of equilibration even in the heparinized state. However, the aforementioned casuistic reports on disturbing pinhole bleeding at the collar site or severe diffuse oozing through the fabric prompted us to restudy the new device in a standardized *in vitro* set up.

There are three main observations from the current study: first, scanning electron microscopic orientation did not show any difference in suture hole size at the collar anastomotic sites compared with the decade-long proven blood impermeability of the EO Plus fabric (3) and the EO-NEO structure (Figure 1). This was also proven for the transluminal orthograde perfusion of the straight EO NEO, regardless of the suture material applied or the standard vs. hemostatic suture fixation of the collar during 60-min perfusion with heparinized porcine blood at 120 mmHg. The oozing volumes through the fabrics, ranging between 0.0026 and 0.0033 ml/cm^2^/min, were negligible and certainly do not play a role in the clinical field (Figure 6 and Table 1A). In the ante-retrograde perfusion test series via the side port, again there was no significant difference in blood loss over the 60-min test period, regardless of whether the collar was attached to the crimped (arch portion of the graft) or to the non-crimped proximally tapered stented portion, irrespective of the suture fixation technique. The resulting blood loss between 37 and 80 ml over 60 min in the fully heparinized state is not clinically relevant (>0.05, Figure 7 and Table 2). However, in series 3, unphysiological stress testing only of the expanded collar region with 10 N for targeted identification of a potential weak spot revealed a significant difference between standard suture line fixation and hemostatic anastomoses with 15 stitches per 1 cm (Figure 8 and Table 3). This finding explains the few personal casuistic reports regarding continuous pinhole bleeding at the collar anastomotic sites, probably associated with a large periaortic space and enormous back bleeding during reperfusion. For prevention of this bleeding problem, two surgical approaches are recommended: first, the avoidance of a hollow space between the proximal stented portion of the EO-NEO and the surrounding aortic tissue. Trimming of the collar to a seam height of 5 mm for a tight anastomosis with the surrounding aortic tissue, reinforced by a close-fitting fixed Teflon strip outside, ensures the blood tightness at the anastomotic site. In case of the inevitable hollow space around the prosthesis, with large backflow and stress onto the suture line, as expected in aneurysms or dissections with a large distal false lumen, this problem can be overcome through a v-shaped resection of the pathological aortic tissue and a provisional double row 3–0 polypropylene suture to achieve a close proximity of the aortic tissue to the EO-NEO. Then, placement of wide surrounding 3–0 polypropylene continuous suture bites on the Teflon-bolstered approximated aortic tissue through the stented graft portion to the arch part of the prosthesis, ignoring the collar, will result in absolute blood tightness, as experienced with the classic EOP for many years (2). Second, another efficient surgical but more time consuming approach is to position additional Teflon pledged 5–0 U-stitches, secured by the application of BioGlue, as propagated by the Hongkong and Seoul groups (14, 15).

In addition to the surgical aspects of this complex surgery, we have often had to deal with intraoperative hyperinflammation, metabolic changes, and hypothermia that lead to bleeding complications. In particular, the hemostasis system and coagulation cascade might be impaired by (deep) hypothermia and the cardiopulmonary bypass itself (13, 16–19). In addition to these mandatory but potentially harmful intraoperative prerequisites, the coagulation system could also be significantly influenced or impaired in patients treated with P2Y_12_ inhibitors or direct oral anticoagulants (DOACs) (20–22). In some patients, an aortic syndrome or dissection might be misdiagnosed as an acute coronary syndrome being loaded with potent P2Y_12_ inhibitors such as ticagrelor. Moreover, the current increased prescription of DOACs, e.g., in chronic atrial fibrillation, has increased bleeding complications in urgent cardio-surgical patients without the recommended washout period (23–26). To overcome and attenuate these potentially harmful effects of hyperinflammation, coagulation, and antithrombotic effects, intraoperative hemoadsorption, e.g., using the CytoSorb® hemoadsorption device, has been introduced into the clinical routine for complex cardiosurgical cases, both intraoperatively and postoperatively (27–34). Mehta et al. (35) showed significantly improved postoperative hemodynamics in aortic surgery patients by applying intraoperative hemoadsorption. Another large propensity score-matched pairs analysis (168 vs. 168 patients) from Munich, Germany, demonstrated that the use of intraoperative hemoadsorption during aortic surgery was associated with a significant reduction in the intraoperative transfusion of blood products, a more stable acid-base balance, and a lower requirement for vasopressors (36). Moreover, it has been shown that in the worst-case scenario of operating on emergent aortic surgery patients with aortic dissections under antithrombotic medication, intraoperative antithrombotic removal by hemoadsorption is effective and results in significantly less bleeding events, blood product use, and surgical re-explorations. Accordingly, a recently published review recommended antithrombotic removal in the cardiovascular surgery setting (37). The removal of direct factor Xa inhibitors, such as rivaroxaban and apixaban, during cardiopulmonary bypass can also be monitored by thromboelastometry (38). This new opportunity for intraoperative antithrombotic drug removal has now also been added to and recommended by the updated intraoperative guidelines of the European Society of Anaesthesiology and Intensive Care (39).

Regarding the serious questions raised by Czerny et al. (5) of continuous oozing through the graft pores in three cases operated on in Freiburg, Germany, and Bologna, Italy, even after protamine application, the hemostatic therapy required huge amounts of red blood cell concentrate, fresh frozen plasma, and platelet transfusion, but not cryoprecipitate or fibrinogen concentrate based on the thromboelastometry results. Accordingly, coagulopathic bleeding cannot be excluded as a reason for the excessive oozing, as reported in this case series (40–42). Bleeding and transfusion of red blood cells and “yellow blood products” such as fresh frozen plasma and platelets, are well known risk factors for increased morbidity [transfusion-associated circulatory overload (TACO), transfusion-associated lung injury (TRALI), and transfusion-related immunomodulation (TRIM) with nosocomial infection and multiple organ failure (MOF)] and mortality in major cardiovascular surgery and other clinical set ups (18, 43).

### Limitations

We believe that the use of porcine heparinized blood for *in vitro* blood permeability testing of the new EO NEO hybrid graft was a successful surrogate for human blood, as shown by an ACT beyond 500 s throughout the experiments. This was required because the COVID-19 lockdown resulted in a scarcity of human blood. Owing to the stability of the results achieved with sample sizes of only four to five, it seemed appropriate not to perform more experiments, especially in light of the production controls applied by the manufacturer for the quality control of the fabric in regard to homogeneous weaving and the placement of branch attachments using hemostatic suture techniques.

### In conclusion

**Figure 9:**
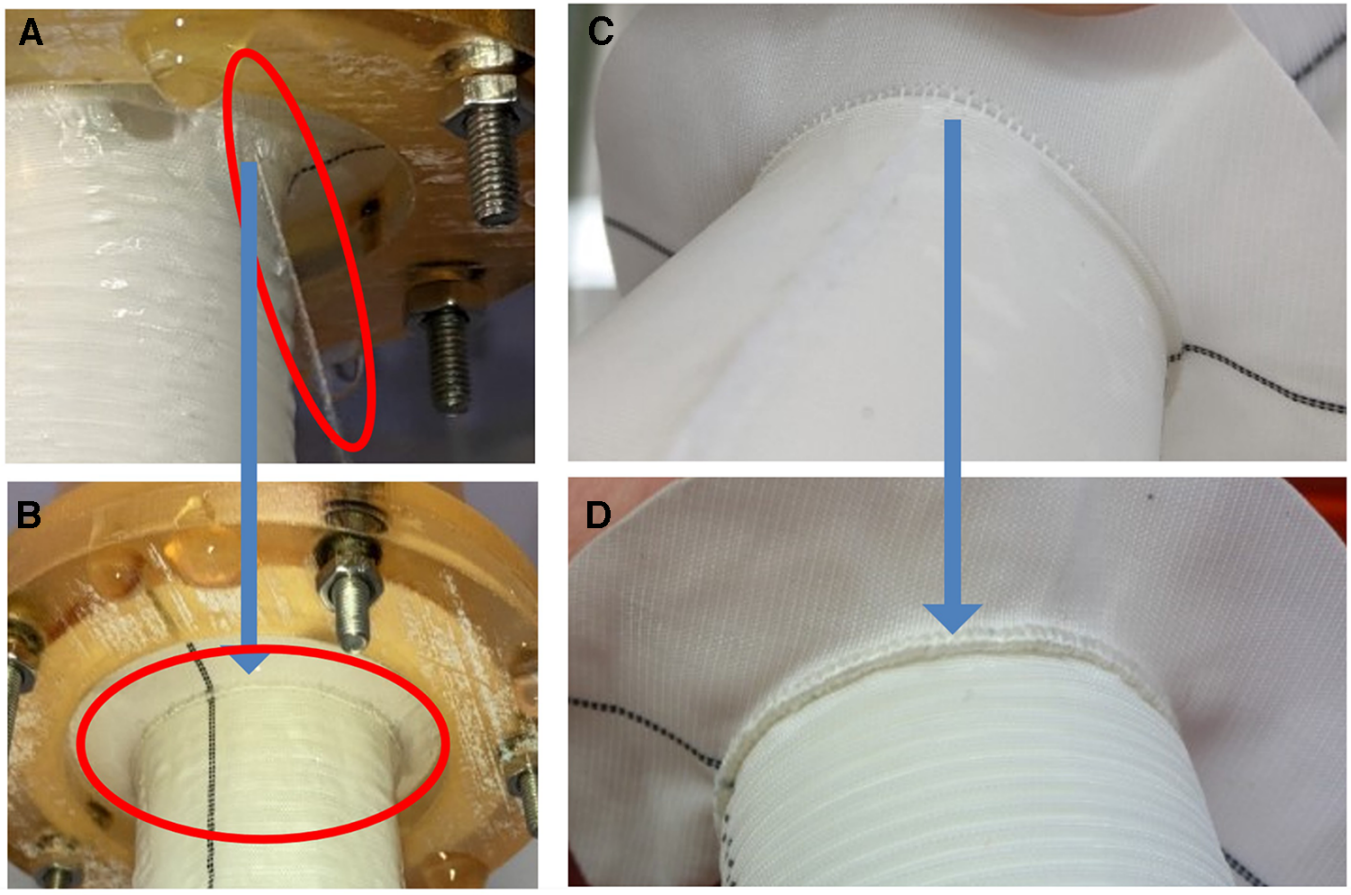
Consequences of the *in vitro* test series (**A–D**). Pinhole leakage (red ellipse) during pre-experiment pressurized saline testing of the standard suture line (**A**) Optimization of standard suture lines (**A,C**) to the hemostatic suture line (**B,D**, blue arrows) using flow weave yarn. (**B**) the red circle demonstrates the absolute water tightness of the collar suture line post correction.

Even in worst case scenarios such as Penn Class BC dissection and/or devastating preoperative conditions of the patient, the combination of rapid surgery and the described optimized surgical techniques, as well as optimized coagulation treatment with cryoprecipitate, fibrinogen concentrate, and prothrombin complex concentrate guided by thromboelastometry, is promising improved results. The anecdotally reported pinhole bleeding instances have been corrected meanwhile by the manufacturer by modifying the standard anastomotic suture line at the collar to a hemostatic one using FWY (Figure 9). The results of the *in vitro* tests clearly demonstrated the blood tightness of the EO-NEO grafts in different set ups, thus the principle of the tightly woven non-covered polyester graft design can be maintained. Excellent clinical results that rely on the aforementioned principles with few instances of perioperative bleeding can be achieved, as demonstrated at Essen University Hospital, Germany, and Ewha Womans University Aorta and Vascular Hospital, South Korea, as well as by other groups.

## Data Availability

The raw data supporting the conclusions of this article will be made available by the authors, without undue reservation.
